# Serum direct bilirubin predicts severe tricuspid regurgitation in atrial fibrillation

**DOI:** 10.3389/fcvm.2025.1691962

**Published:** 2025-12-05

**Authors:** Xin-yue Zou, Da-wei Lin, Zhao-xia Wang, Chen Wu, Da-xin Zhou, Chun-yan Shi, Yao-sheng Wang

**Affiliations:** 1 Department of Cardiology, Xinhua Hospital Affiliated to the Shanghai Jiao Tong University School of Medicine, Shanghai, China; 2 Department of Cardiology, Zhongshan Hospital Affiliated to the Fudan University School of Medicine, Shanghai, China; 3 Department of Cardiology, Shanghai Pudong New Area Zhoupu Hospital (Shanghai Health Medical College Affiliated Zhoupu Hospital), Shanghai, China; 4 Department of Cardiology, Renji Hospital Affiliated to the Shanghai Jiao Tong University School of Medicine, Shanghai, China; 5 Department of Information, Chongming Hospital Afflicted to Shanghai University of Medicine and Health Sciences, Shanghai, China; 6 Clinical Research and Innovation Unit, Xinhua Hospital Affiliated to Shanghai Jiao Tong University School of Medicine, Shanghai, China

**Keywords:** moderate-to-severe tricuspid regurgitation, atrial fibrillation, direct bilirubin, antibiotic use, microbial translocation

## Abstract

**Aims:**

Moderate-to-severe tricuspid regurgitation (TR) often coexists with atrial fibrillation (AF) and is associated with poor prognoses. Although inflammation is elevated in TR patients, whether bilirubin predicts moderate-to-severe TR in AF remains unclear. This study aims to evaluate serum bilirubin as an early diagnostic marker for TR in AF.

**Method and results:**

We enrolled 344 AF patients between 2020 and 2023 and collected clinical data, including diagnoses, blood tests, medication history, and transthoracic echocardiography parameters. Patients were grouped by TR severity (AF with vs. without moderate-to-severe TR) or antibiotic use (users vs. non-users). After adjusting for confounders, univariate and multivariate Cox regression analyses were performed to gauge variable associations with TR occurrence. A receiver operating characteristic (ROC) analysis assessed the predictive accuracy of direct bilirubin (DBIL) for TR, and Kaplan–Meier curves depicted the cumulative 48-month TR incidence in patients with DBIL >3.5 µmol/L. Patients with moderate-to-severe TR had higher DBIL (5 vs. 3 µmol/L, *p* < 0.001), direct bilirubin, B-type natriuretic peptide, and *γ*-GGT values. A multivariate Cox regression showed that elevated DBIL independently predicted TR (HR = 1.104, *p* = 0.039). The ROC analysis identified DBIL ≥3.5 µmol/L as an optimal cutoff for distinguishing TR (AUC = 0.846, sensitivity 91.2%, specificity 68.8%). Among patients with DBIL >3.5 µmol/L, antibiotic use reduced TR risk (HR = 0.214, *p* < 0.001).

**Conclusions:**

Serum DBIL levels serve as a potential early diagnostic marker for moderate-to-severe TR in AF patients, and anti-infective therapy lowers TR incidence.

## 1 Introduction

Atrial fibrillation (AF) is a highly prevalent arrhythmia affecting millions globally (1, 2), inducing aberrant atrial electrical activity and impairing systolic function, which elevates risks of heart disease and stroke (3, 4). The robust association between AF and multiple cardiovascular events underscores its significant clinical burden, establishing it as a major public health concern. Tricuspid regurgitation (TR), characterized by pathological backflow of blood into the right atrium during ventricular contraction, is stratified by severity into mild, moderate, or severe (5, 6). This association of valvulopathy with AF is particularly clinically relevant since it exacerbates cardiac structural remodeling and functional impairment. Persistent AF frequently predisposes to moderate-to-severe TR, consequently elevating risks of heart failure and mortality (7). In population-based studies, nearly one-third of AF cohorts progress to clinically significant TR, strongly correlating with poor survival (8), necessitating predictive biomarkers and preventive interventions.

Emerging evidence indicates that serum direct bilirubin (DBIL) may serve as a promising biomarker for certain cardiovascular disorders (9). While traditionally recognized as a marker of hepatic function and heme catabolism, DBIL has demonstrated prognostic utility in conditions such as right heart failure. Nevertheless, its specific role in detecting moderate-to-severe TR among patients with AF remains inadequately characterized.

The pathophysiological milieu in AF patients is frequently complicated by a state of chronic, low-grade systemic inflammation (10). This inflammatory environment is thought to stem from multifactorial sources, including immune dysregulation, therapeutic interventions, and underlying comorbidities (11). Such proinflammatory conditions are known to exacerbate the progression of cardiovascular pathologies, as demonstrated in studies of complex disease mechanisms (12).

A plausible mechanistic link between inflammation and DBIL elevation may involve dysfunction of the gut–heart axis (13). Theoretical frameworks suggest that inflammatory mediators, potentially derived from gut microbiota, could translocate via enterohepatic circulation, consistent with observations of multifactorial pathogenic processes in other clinical contexts. This process might concurrently induce hepatic inflammation, perturb bilirubin metabolism, and contribute to low-grade cardiac tissue injury, mirroring patterns seen in infectious complications affecting cardiac structures (13).

Building upon this conceptual foundation, we propose a novel hypothesis wherein a subset of AF patients may experience a cycle of bacterially driven inflammation—potentially amplified by therapeutic agents and immune alterations—that could facilitate the translocation of inflammatory mediators or microbial products (14). This cascade is hypothesized to initiate parallel pathophysiological events including hepatic stress and/or erythrocyte lysis, leading to elevated serum DBIL levels. In addition, persistent, low-grade injury to right heart structures may promote structural degradation and progression to moderate-to-severe TR, reflecting the complex interplay of factors observed in various cardiovascular risk conditions.

Consequently, elevated DBIL in this context may not merely represent a passive biomarker but could reflect the activity of this shared inflammatory pathway. If this mechanism is operative, targeted antibiotic therapy could theoretically attenuate the process by modulating gut microbiota and reducing bacterial inflammatory burden, thereby potentially mitigating valvular pathogenesis in AF patients with elevated DBIL levels, analogous to approaches used in managing infectious complications in cardiac contexts.

## 2 Materials and methods

### 2.1 Study participants

Our study retrospectively analyzed electronic medical records of patients diagnosed with AF and moderate-to-severe TR who received treatment at the Chongming Branch of Xin Hua Hospital between 2020 and 2023. Clinical data were meticulously collected and analyzed from the hospital's electronic medical records using the Full-Lifecycle Group Health Management Data Integration Cloud Platform. These data included initial admission diagnoses, comprehensive blood examination results, and detailed parameters from transthoracic echocardiography (TTE), enabling a thorough evaluation of cardiac condition progression and management.

A total of 344 patients were included: 253 with AF without moderate-to-severe TR and 91 with AF and moderate-to-severe TR. Exclusion criteria consisted of (1) severe liver disease, (2) hemolytic disease, (3) biliary disease, (4) severe pulmonary disease, (5) congenital heart disease, (6) active infective endocarditis, (7) active tuberculosis, and (8) conditions requiring long-term specialized antibiotic regimens (e.g., chronic osteomyelitis with ongoing suppressive therapy).

### 2.2 Antibiotic use definition and group classification

Patients were classified into an antibiotic user group if they received ≥3 consecutive days of oral antibiotic therapy between 30 days prior to admission and the date of discharge. Antibiotics administered included β-lactams (e.g., amoxicillin/clavulanate, cefuroxime, or cefixime), macrolides (e.g., azithromycin or clarithromycin), and fluoroquinolones (e.g., levofloxacin or moxifloxacin). Single-dose or prophylactic antibiotic use lasting ≤24 h was not classified as antibiotic exposure. All antibiotic courses were within a 1-month time frame. Indications for antibiotic treatment comprised bacterial infections of the lower respiratory tract, upper respiratory tract, urinary tract, skin and soft tissue, oral/dental sources, and gastrointestinal system.

### 2.3 Ethical considerations

The study protocol was approved by the local ethics committee. Because of the retrospective and observational nature of the study, the requirement for informed consent was waived. All patient data were anonymized prior to analysis. Individual clinical information was accessible only through inpatient numbers derived from participant identification codes, ensuring confidentiality. This study adhered to the principles of the Declaration of Helsinki (2013 revision).

### 2.4 Collection of blood biomarker examination outcomes

Serum biomarkers were quantified from venous blood samples collected in separator tubes (centrifuged at 3,000×g for 10 min). Clinical data were extracted for the following parameters: total bilirubin (TBIL) and DBIL, measured by diazo colorimetry (detection limit 0.1 mg/dL); B-type natriuretic peptide (BNP), assessed via chemiluminescent microparticle immunoassay (ARCHITECT analyzer, Abbott); γ-glutamyltranspeptidase (γ-GGT), alanine aminotransferase (ALT), and aspartate aminotransferase (AST), measured using enzymatic rate methods (Roche Cobas c501); albumin (ALB), analyzed by bromocresol green binding (sensitivity 0.1 g/dL); globulin (GLB) and total protein (TP), assessed via biuret reaction; and albumin/globulin ratio (A/G), calculated from these measurements. All parameters were analyzed in accordance with CLSI guideline EP09-A3.

### 2.5 Transthoracic echocardiography

Transthoracic echocardiography was performed with patients in the left lateral decubitus position to optimize acoustic windows, acquiring comprehensive views for structural and functional assessment. Measurements were averaged over three consecutive cardiac cycles for sinus rhythm patients and five cycles for atrial fibrillation cases. All studies were performed in a blinded manner by two experienced cardiologists (>5-year TTE specialization), with interobserver discrepancies resolved through consensus.

### 2.6 Statistical analysis

Continuous variables were expressed as mean ± standard deviation for normally distributed data and as median (interquartile range) for non-normally distributed data. Categorical data were presented as percentages. Statistical differences between groups were analyzed using an unpaired Student's *t*-test and a *χ*^2^ test. Cox proportional models were applied to calculate the hazard ratio (HR) and 95% confidence interval (CI) to evaluate the association of serum DBIL and oral antibiotics with moderate-to-severe TR in AF patients. Kaplan–Meier curves and the log-rank test were employed to compare the moderate-to-severe TR-free survival in antibiotic users and non-users. Analyses were performed using Stata17, and scatter diagrams were generated using GraphPad Prism 9.5.1. A *p*-value of less than 0.05 was considered statistically significant.

## 3 Results

### 3.1 Baseline and clinical characteristics of patients with AF with or without moderate-to-severe TR

This study enrolled 344 AF patients, identifying 91 with moderate-to-severe TR vs. 253 without TR during a median 13.1-month follow-up. Baseline characteristics (Table 1) showed no significant intergroup differences in demographics (sex), comorbidities (hypertension, diabetes, chronic kidney disease, and hyperlipidemia), pharmacotherapy (warfarin, ACE inhibitors, angiotensin receptor blockers, β-blockers, calcium channel blockers, stains, and clopidogrel), or liver function markers (ALT, AST, albumin/globulin (A: G) ratio, and total protein). The moderate-to-severe TR group demonstrated significantly higher values for age [80 (74–84) vs. 76 (69–83) years, *p* = 0.009], BNP [403 (246–575) vs. 210 (98–368) pg/mL, *p* < 0.001], total bilirubin [21 (16–28) vs. 16 (13–20) μmol/L, *p* < 0.001], direct bilirubin [5 (4–7) vs. 3 (2–4) μmol/L, *p* < 0.001], TR peak gradient [55 (46–63) vs. 41 (36–44) mmHg, *p* < 0.001], and pulmonary artery systolic pressure [62 (54–72) vs. 54 (45–54) mmHg, *p* < 0.001]. Notably, among 158 patients with elevated bilirubin, antibiotic therapy (*n* = 48, 30.4%) was associated with substantially lower moderate-to-severe TR incidence [five cases (5.5%) vs. expected 30.4%, *p* < 0.001], with detailed characteristics provided in Table 2.

**Table 1:** Baseline and clinical characteristics of patients with AF with and without moderate-to-severe TR.

Variables	AF without moderate-to- severe TR *n* = 253	AF with moderate-to- severe TR *n* = 91	*P*-value
Age, years	76 (69, 83)	80 (74, 84)	0.009
Male (%)	151 (59.7)	54 (59.3)	0.95
RFCA (%)	27 (10.7)	11 (12.1)	0.71
AF (%)			
Paroxysmal	178 (70.4)	35 (38.5)	<0.001
Persistent	67 (26.5)	55 (60.4)	
Permanent	8 (3.2)	1 (1.1)	
HBP (%)	239 (94.5)	85 (93.4)	0.71
NYHA (%)			
Grade I	149 (58.9)	33 (36.3)	
Grade II	50 (19.8)	20 (22.0)	<0.001
Grade III	34 (13.4)	21 (23.1)	
Grade IV	20 (7.9)	17 (18.7)	
DM (%)	4 (1.6)	1 (1.1)	0.74
CKD (%)	80 (31.6)	37 (40.7)	0.12
HL (%)	101 (39.9)	43 (47.3)	0.22
Drugs using			
Warfarin (%)	224 (88.5)	80 (87.9)	0.87
β-blocker (%)	236 (93.3)	84 (92.3)	0.75
CCB (%)	218 (86.2)	80 (87.9)	0.67
ACEI (%)	10 (4)	4 (4.4)	0.85
ARB (%)	229 (90.5)	82 (90.1)	0.91
Antibiotic using (%)	76 (30.0)	17 (18.7)	0.036
Statins (%)	64 (25.3)	19 (20.9)	0.40
Clopidogrel (%)	41 (16.2)	8 (8.8)	0.083
TTE			
Grade of MR (%)			
None	8 (3.2)	0 (0)	
Mild	215 (85.0)	57 (62.6)	<0.001
Moderate	27 (10.7)	28 (30.8)	
Severe	3 (1.2)	6 (6.6)	
PASP, mmHg	54 (45, 54)	62 (54, 72)	<0.001
Blood examination			
BNP, pg/mL	210 (98, 368)	403 (246, 575)	<0.001
TBIL, µmol/L	16 (13, 20)	21 (16, 28)	<0.001
DBIL, µmol/L	3 (2, 4)	5 (4, 7)	<0.001
ALT, µ/L	12 (8, 17)	11 (8, 15)	0.89
AST, µ/L	24 (20, 31)	27 (23, 36)	0.033
*Γ*-GGT, µ/L	30.5 (20, 55)	42 (26, 66)	<0.001
ALB, µ/L	39 (36, 42)	37.5 (34, 42)	0.11
GLB, g/L	29 (26, 32)	28 (26, 31)	0.73
A/G	1.34 (1.18, 1.52)	1.31 (1.15, 1.52)	0.39
TP, g/L	68 (64.5, 71)	67 (62, 72)	0.17

Data are presented as mean ± standard deviation or number (%) of patients. Non-normally distributed data are shown as median (25th–75th percentile).

AF, atrial fibrillation; TR, tricuspid regurgitation; MR, mitral regurgitation; RFCA, radio-frequency catheter ablation; HBP, high blood pressure; DM, diabetes mellitus; CKD, chronic kidney disease; HL, hyperlipidemia; CCB, calcium channel block; ACEI, angiotensin-converting enzyme inhibitor; ARB, angiotensin receptor blocker; PASP, pulmonary artery systolic pressure; BNP, brain natriuretic peptide; DBIL, direct bilirubin; TBIL, total bilirubin; γ-GGT, γ-glutamyltranspeptidase; ALT, alanine aminotransferase; AST, aspartate aminotransferase; ALB, albumin; GLB, globulin; A/G, albumin/globulin; TP, total protein.

**Table 2:** Baseline and clinical characteristics of antibiotic users and non-users.

Variable	Non-users *n* = 251	Antibiotic users *n* = 93	*P*-value
Age, years	78 (71, 84)	76 (68, 81)	0.021
Male (%)	149 (59.4)	56 (60.2)	0.89
RFCA (%)	31 (12.4)	7 (7.5)	0.20
AF (%)			
Paroxysmal	156 (62.2)	57 (61.3)	
Persistent	89 (35.5)	33 (35.5)	0.86
Permanent	6 (2.4)	3 (3.2)	
HBP (%)	235 (93.6)	89 (95.7)	0.47
NYHA (%)			
Grade I	131 (52.2)	51 (54.8)	
Grade II	53 (21.1)	17 (18.3)	0.76
Grade III	42 (16.7)	13 (14.0)	
Grade IV	25 (10.0)	12 (12.9)	
DM (%)	3 (1.2)	2 (2.2)	0.51
CKD (%)	85 (33.9)	32 (34.4)	0.92
HL (%)	98 (39.0)	46 (49.5)	0.082
Drugs using			
Warfarin (%)	222 (88.4)	82 (88.2)	0.94
β-blocker (%)	233 (92.8)	87 (93.5)	0.82
CCB (%)	217 (86.5)	81 (87.1)	0.88
ACEI (%)	12 (4.8)	2 (2.2)	0.27
ARB (%)	224 (89.2)	87 (93.5)	0.23
Moderate-to-severe TR (%)	74 (29.5)	17 (18.3)	0.036
Statins (%)	60 (23.9)	23 (24.7)	0.87
Clopidogrel (%)	37 (14.7)	12 (12.9)	0.66
TTE			
Grade of MR (%)			
None	7 (2.8)	1 (1.1)	
Mild	198 (78.9)	74 (79.6)	0.85
Moderate	40 (15.9)	15 (16.1)	
Severe	6 (2.4)	3 (3.2)	
PASP, mmHg	54 (47, 58)	54 (48, 57)	0.70
Blood Examination			
BNP, pg/mL	237 (134, 484)	281 (100, 484)	0.67
TBIL, µmol/L	16 (13, 21)	19 (14, 23)	0.007
DBIL, µmol/L	3 (2, 5)	4 (2.5)	0.002
ALT, µ/L	12 (8, 16)	11 (8, 18)	0.78
AST, µ/L	25 (20, 33)	25 (19, 32)	0.37
*Γ*-GGT, µ/L	34 (21, 57)	31 (21, 57)	0.87
ALB, µ/L	39 (36, 42)	39 (35, 42)	0.80
GLB, g/L	29 (26, 32)	29 (26, 32)	0.71
A/G	1.33 (1.16, 1.52)	1.34 (1.2, 1.57)	0.42
TP, g/L	68 (64, 71)	68 (63, 71)	0.89
Follow-up, months	12.3(3.5, 22.8)	14.7(7.5, 26.1)	0.061

Data are presented as mean ± standard deviation or number (%) of patients. Non-normally distributed data are shown as median (25th–75th percentile).

AF, atrial fibrillation; TR, tricuspid regurgitation; MR, mitral regurgitation; RFCA, radio-frequency catheter ablation; HBP, high blood pressure; DM, diabetes mellitus; CKD, chronic kidney disease; HL, hyperlipidemia; CCB, calcium channel block; ACEI, angiotensin-converting enzyme inhibitor; ARB, angiotensin receptor blocker; TR, tricuspid regurgitation; PASP, pulmonary artery systolic pressure; BNP, brain natriuretic peptide; DBIL, direct bilirubin; TBIL, total bilirubin; γ-GGT, γ-glutamyltranspeptidase; ALT, alanine aminotransferase; AST, aspartate aminotransferase; ALB, albumin; GLB, globulin; A/G, albumin/globulin; TP, total protein.

### 3.2 Baseline and clinical characteristics of antibiotic users and non-users

Among 344 AF patients (93 antibiotic users, 251 non-users), baseline characteristics were largely comparable, except for key differences. Antibiotic users were younger [median age 76 (68–81) vs. 78 (71–84) years, *p* = 0.021] and exhibited a trend toward higher hyperlipidemia prevalence (49.5% vs. 39.0%, *p* = 0.082), while demonstrating significantly lower moderate-to-severe tricuspid regurgitation incidence (18.3% vs. 29.5%, *p* = 0.036). Biochemically, users had elevated bilirubin levels [TBIL: 19 (14–23) vs. 16 (13–21) μmol/L, *p* = 0.007; DBIL: median 4 vs. 3 μmol/L, *p* = 0.002] but comparable values across other hepatic markers. No significant differences were observed in terms of gender distribution, ablation rates, AF subtypes, comorbidities (hypertension, diabetes, renal disease), cardiovascular medications, echocardiographic parameters (mitral regurgitation severity, PASP), or additional blood biomarkers.

### 3.3 Serum biomarkers between groups

**Figure 1:**
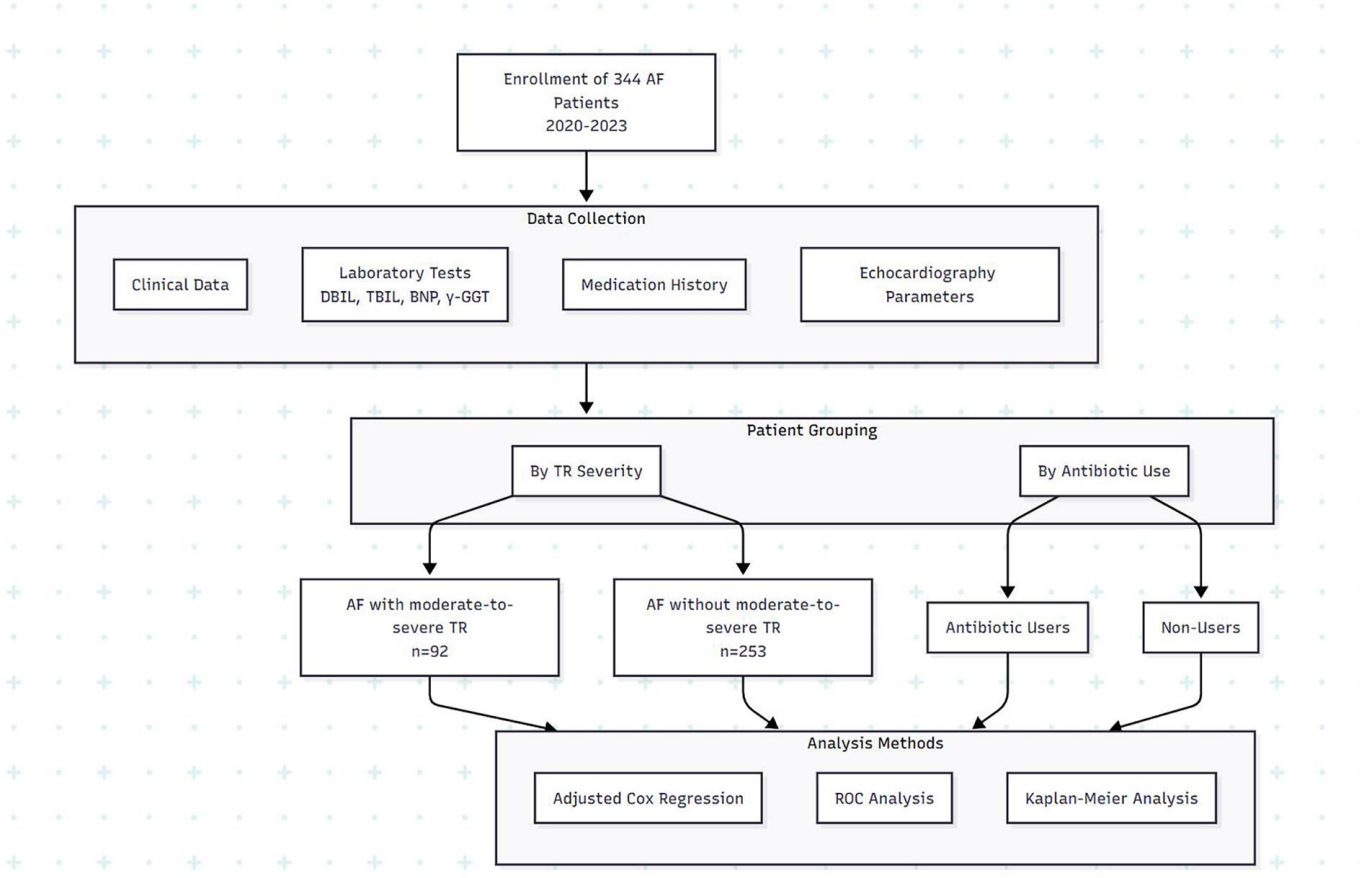
Flowchart of the present study.

Table 1 presents serum biomarker data. Levels of DBIL [5 (4–7) vs. 3 (2–4) μmol/L, *p* < 0.001], TBIL [21 (16–28) vs. 16 (13–20) μmol/L, *p* < 0.001], BNP [403 (246–575) vs. 210 (98–368) pg/mL, *p* < 0.001], and γ-GGT [30.5 (20–55) vs. 42 (26–66) µ/L, *p* < 0.001] were significantly higher in AF patients with moderate-to-severe TR compared with those without. In addition, AST levels were higher in AF patients with moderate-to-severe TR [24 (20–31) vs. 27 (23–36), *p* = 0.033]. Graphical representations of the blood indices are provided in Figure 1.

### 3.4 Association of serum biomarkers with moderate-to-severe TR among patients with AF

To investigate the relationship between various parameters and moderate-to-severe TR in patients with AF, we conducted a Cox regression analysis. Single-variable Cox regression revealed that age, antibiotic using, MR, AF type, NYHA classification, PASP, BNP, TBIL, and DBIL were significantly associated with moderate-to-severe TR in AF patients. Further multivariate Cox regression analysis demonstrated that antibiotic usage (HR = 0.275; 95% CI: 0.104, 0.726; *p* = 0.009) and serum DBIL (HR = 1.135, 95% CI: 1.038, 1.241; *p* = 0.006) remained significantly associated with moderate-to-severe TR after adjusting for confounders, including age, MR, AF type, NYHA classification, PASP, TBIL, and BNP.

### 3.5 Serum DBIL level for predicting moderate-to-severe TR in AF patients

Table 3 presents the results of single-variable and multivariable Cox regression analyses, examining the association between various clinical variables and disease outcomes. In the single-variable analysis, several factors show significant associations with disease outcomes, including age (HR = 1.026, *p* = 0.043), antibiotic use (HR = 0.548, *p* = 0.026), pulmonary artery systolic pressure (PASP) (HR = 1.053, *p* < 0.001), moderate or severe mitral regurgitation (MR) (HR = 2.369, *p* < 0.001), persistent (HR = 3.040, *p* < 0.001) and permanent (HR = 0.856, *p* = 0.879) AF, New York Heart Association (NYHA) functional class II (HR = 1.391, *p* = 0.246), NYHA III (HR = 1.938, *p* = 0.018), NYHA IV (HR = 3.558, *p* < 0.001), brain natriuretic peptide (BNP) (HR = 1.000, *p* = 0.002), TBIL (HR = 1.059, *p* < 0.001), DBIL (HR = 1.174, *p* < 0.001), and γ-glutamyltranspeptidase (γ-GGT) (HR = 1.003, *p* = 0.085). However, in the multivariable analysis, only some of these variables maintain statistical significance, such as PASP (HR = 1.036, *p* < 0.001), DBIL (HR = 1.104, *p* = 0.039), and certain categories of AF and NYHA class, suggesting that these factors independently predict disease outcomes after adjusting for other variables.

**Table 3:** Single-variable and multivariable Cox regression analyses of association between variables and disease outcome status.

Variable	Single-variable		Multivariable	
	HR value	*P*-value	HR value	*P*-value
Age	1.026	**0**.**043**	1.018	0.220
Antibiotic using	0.548	**0**.**026**	0.566	**0.044**
PASP	1.053	**<0**.**001**	1.036	<0.001
Grade of MR				
None or Mild	–	–	–	–
Moderate or Severe	2.369	**<0.001**	1.237	0.418
Classification of AF				
Paroxysmal	–	–	–	–
Persistent	3.040	**<0.001**	2.416	**0.001**
Permanent	0.856	0.879	0.845	0.869
NYHA				
Grade I	–	–	–	–
Grade II	1.391	0.246	1.023	0.937
Grade III	1.938	**0.018**	0.729	0.350
Grade IV	3.558	**<0.001**	1.547	0.212
BNP	1.000	**0**.**002**	1.000	0.543
TBIL	1.059	**<0**.**001**	0.997	0.892
DBIL	1.174	**<0**.**001**	1.104	**0.039**
AST	1.005	0.337	–	–
*Γ*-GGT	1.003	0.085	–	–

AF, atrial fibrillation; TR, tricuspid regurgitation; MR, mitral regurgitation; RFCA, radio-frequency catheter ablation; PASP, pulmonary artery systolic pressure; BNP, brain natriuretic peptide; DBIL, direct bilirubin; TBIL, total bilirubin; γ-GGT, γ-glutamyltranspeptidase; AST, aspartate aminotransferase.

Bold values indicate statistical significance.

**Figure 2:**
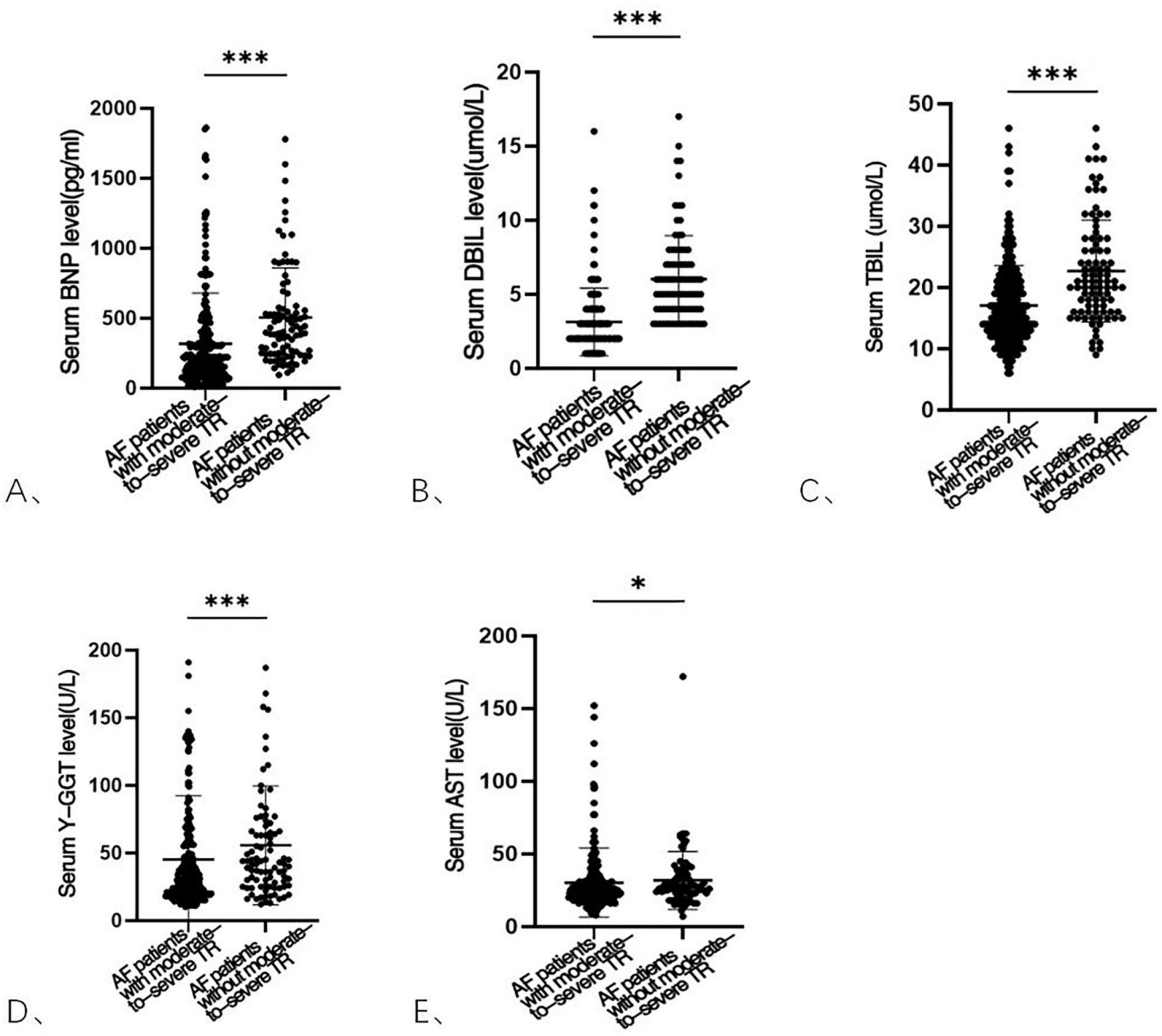
Part serum biomarker levels in AF patients with and without moderate-to-severe TR. BNP, brain natriuretic peptide **(A)**; DBIL, direct bilirubin **(B)**; TBIL, total bilirubin **(C)**; γ-GGT, γ-glutamyltranspeptidase **(D)**; AST, aspartate aminotransferase **(E)**. AF, atrial fibrillation; TR, tricuspid regurgitation.

As shown in Figure 2, a DBIL level of 3.5 µmol/L discriminated AF patients with moderate-to-severe TR from those without moderate-to-severe TR, with a sensitivity of 91.2% and a specificity of 68.8% (AUC: 0.846, 95% CI 0.806–0.887 *p* < 0.001).

**Figure 3:**
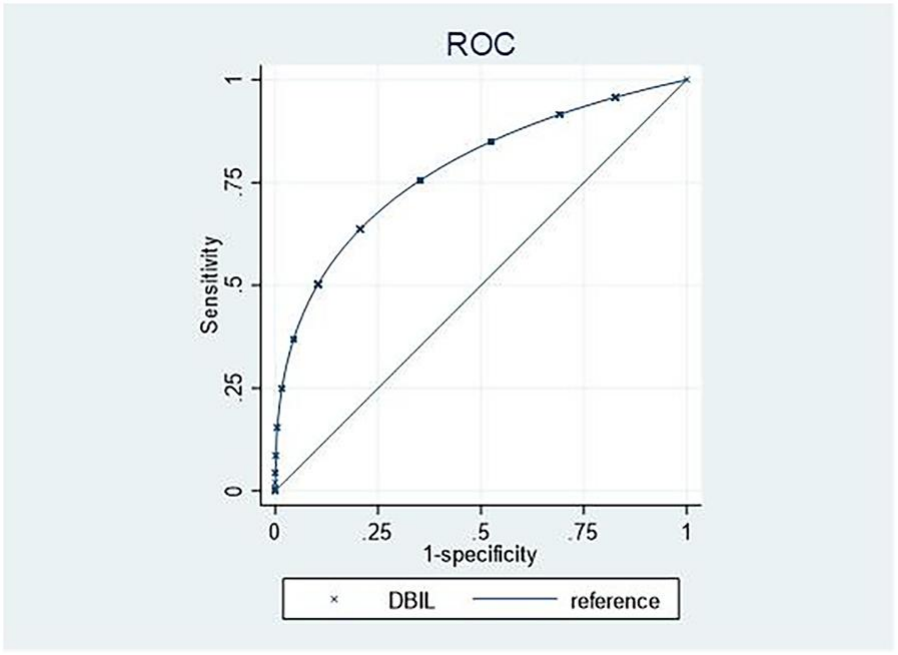
ROC analyses of serum biomarkers predicting AF patients with moderate-to-severe TR. ROC, receiver operating characteristic; AF, atrial fibrillation; TR, tricuspid regurgitation.

**Figure 4:**
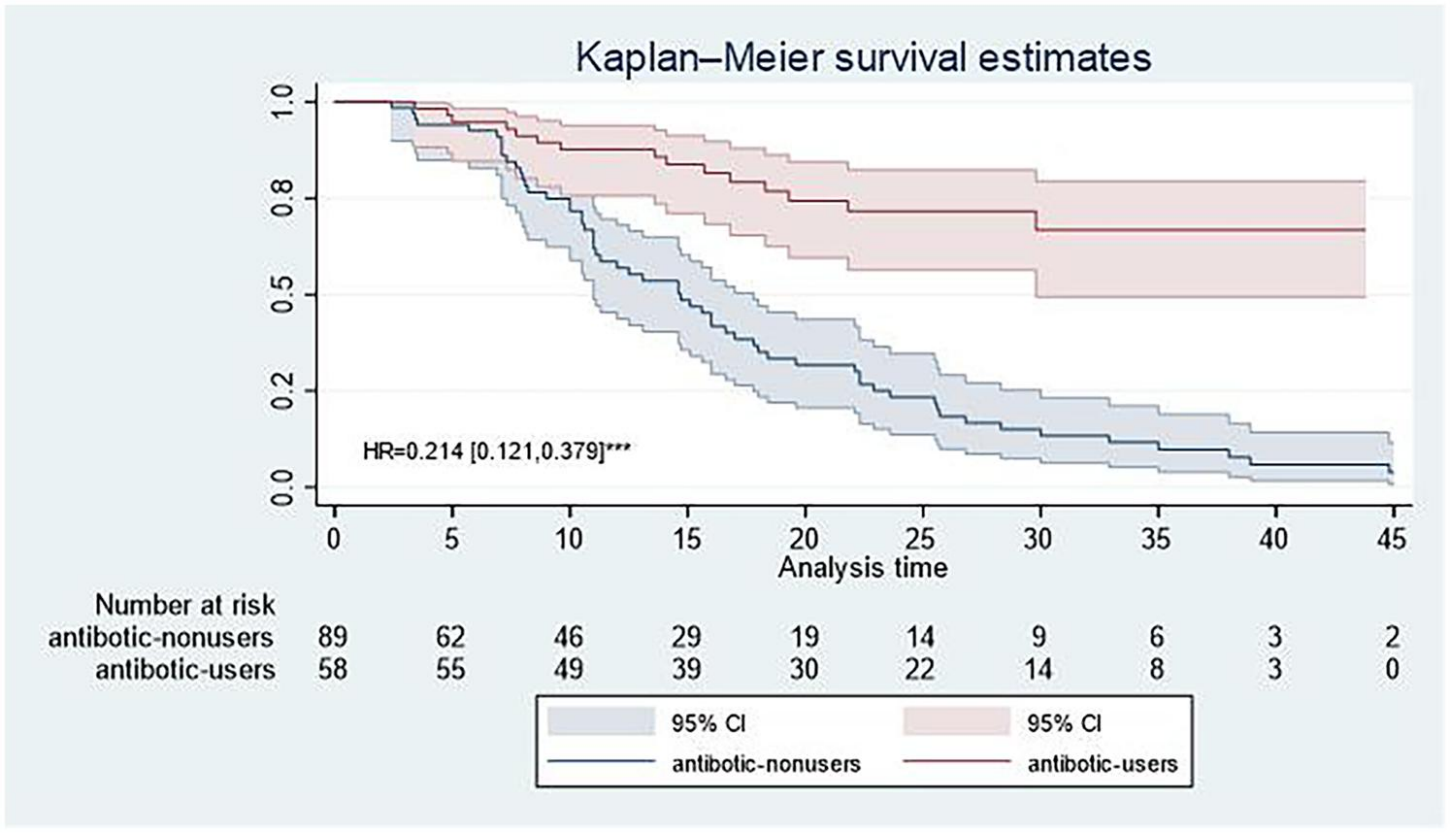
Kaplan–Meier curves showing cumulative 48-month moderate-to-severe TR in AF patients with serum DBIL >3.5 µmol/L.

As shown in Figure 3 and Table 4, we selected patients with serum DBIL values of >3.5 μmol/L and generated Kaplan–Meier curves for their antibiotic use and outcome status during follow-up. The follow-up was conducted through regular outpatient clinic visits, telephonic interviews, and review of electronic medical records, with a median follow-up of 13.1 months. A Kaplan–Meier analysis assessed TR-free survival, defined as freedom from progression to moderate-to-severe TR or TR-related events (e.g., worsening TR severity). Figure 4 illustrates a significant difference in TR-free survival rates between antibiotic users and non-users over a 45-month period (as specified in the figure), with the survival curve for antibiotic users (red line) consistently higher than that for non-users (blue line), indicating a substantial advantage in reducing TR progression. A HR of 0.214 [0.121, 0.379] further underscores this advantage, demonstrating that antibiotic users have a markedly lower risk of TR progression compared with non-users. The curves of both groups start at 1.0 but diverge sharply, with the non-user group declining to approximately 0.1 by the end, while the user group remains around 0.8. The narrow 95% confidence intervals (shaded areas) around each curve reflect high confidence in these estimates. With regard to the potential dynamic relationship between DBIL and TR, data on DBIL levels after TR treatment (e.g., post-surgery) were not available in this retrospective analysis; this could be explored in future studies to establish causality.

**Table 4:** A comparison of variables between antibiotic users and non-users in serum DBIL >3.5 µmol/L AF patients.

Variable	Non-users *n* = 89	Antibiotic users *n* = 58	*P*-value
Age, years	80 (74, 85)	76 (70, 81)	0.008
Male (%)	53 (60)	35 (60)	0.92
Moderate-to-severe TR (%)	59 (66)	15 (26)	<0.001
RFCA (%)	12 (13)	5 (9)	0.37
AF (%)			0.35
Paroxysmal	40 (45)	33 (57)	
Persistent	46 (52)	23 (40)	
Permanent	3 (3)	2 (3)	
HBP (%)	84 (94)	56 (97)	0.55
NYHA (%)			0.42
Grade I	38 (43)	30 (52)	
Grade II	16 (18)	13 (22)	
Grade III	19 (21)	9 (16)	
Grade IV	16 (18)	6 (10)	
DM (%)	1 (1)	1 (2)	0.76
CKD (%)	32 (36)	21 (36)	0.98
HL (%)	40 (45)	32 (55)	0.23
Drugs using			
Warfarin (%)	77 (87)	50 (86)	0.96
β-blocker (%)	233 (92.8)	87 (93.5)	0.82
CCB (%)	217 (86.5)	81 (87.1)	0.88
ACE I (%)	3 (3)	1 (2)	0.55
ARB (%)	82 (92)	55 (95)	0.53
Statins (%)	19 (21)	13 (22)	0.88
Clopidogrel (%)	13 (15)	7 (12)	0.66
TTE			
Grade of MR (%)			
None	3 (3)	0 (0)	0.32
Mild	58 (65)	45 (78)	
Moderate	25 (28)	11 (19)	
Severe	3 (3)	2 (3)	
PASP, mmHg	55 (49, 67)	54 (48, 62)	0.23
Blood examination			
BNP, pg/mL	382 (220, 587)	271 (135, 528)	0.040
TBIL, µmol/L	21 (17, 28)	21.5 (19, 27)	0.82
DBIL, µmol/L	6 (4, 7)	4 (3, 6)	0.002
ALT, µ/L	12 (9, 15)	12.5 (8, 19)	0.79

**Table:** 

AST, µ/L	28 (22, 37)	26.5 (20, 33)	0.40
*Γ*-GGT, µ/L	41 (26, 70)	37 (24, 68)	0.56
ALB, µ/L	38 (34, 41)	38 (35, 42)	0.45
GLB, g/L	28 (26, 31)	28 (26, 32)	0.79
A/G	1.31 (1.14, 1.46)	1.34 (1.18, 1.59)	0.28
TP, g/L	66 (62, 71)	67 (63, 71)	0.71

AF, atrial fibrillation; TR, tricuspid regurgitation; MR, mitral regurgitation; RFCA, radio-frequency catheter ablation; PASP, pulmonary artery systolic pressure; BNP, brain natriuretic peptide; DBIL, direct bilirubin; TBIL, total bilirubin; γ-GGT, γ-glutamyltranspeptidase; AST, aspartate aminotransferase.

## 4 Discussion

AF and TR are pathophysiologically intertwined. AF-induced loss of atrial contractility causes intra-atrial stasis and impaired ventricular diastolic filling, directly increasing right ventricular preload. This promotes tricuspid annular dilatation and leaflet malcoaptation, driving TR initiation and progression (14, 15). As AF persists, progressive TR exacerbates right ventricular volume overload, inducing chamber dilation and functional decompensation. This vicious cycle significantly elevates risks of heart failure and all-cause mortality (HR 1.89, 95% CI 1.45–2.47) (14). Furthermore, TR-mediated venous congestion and reduced cardiac output accelerate end-organ hypoperfusion, particularly worsening renal function in cardiorenal syndrome (16, 17). Consequently, early detection of moderate-to-severe TR in AF patients represents a critical therapeutic window to interrupt this pathophysiological cascade and improve outcomes.

Serum DBIL, a hepatically conjugated heme metabolite processed by uridine diphosphate (UDP)-glucuronosyltransferase (18–20), exerts cardioprotective effects through antioxidant and anti-inflammatory actions. In particular, DBIL suppresses NF-κB signaling and NLRP3 inflammasome activation (21), countering key drivers of atrial fibrillation and thrombosis (22–26). TR pathogenesis arises from interconnected pathways where chronic volume overload induces annular dilation and leaflet malcoaptation, while inflammatory triggers—notably streptococcal endocarditis—promote valvular vegetation formation and chordal rupture through microbial adhesion–mediated tissue destruction. Concurrently, right ventricular dysfunction secondary to pulmonary hypertension displaces papillary muscles, which further compromises coaptation. Elevations in circulating DBIL correlate with TR severity progression as demonstrated in both heart failure cohorts (*r* = 0.62, *p* < 0.001) (27) and congenital cases (+38% vs. controls) (28), thereby positioning DBIL as a biomarker reflective of both TR-induced hemolytic stress from turbulent regurgitant jets and compensatory anti-inflammatory modulation within the aflo-tricuspid apparatus in AF patients.

Emerging evidence indicates that serum DBIL serves as a biomarker for enterohepatic inflammation, reflecting gut-derived endotoxemia and intestinal barrier disruption via the portal circulation. Critically, this inflammatory cascade propagates to the right heart—which exhibits amplified cytokine sensitivity due to 2.5-fold higher baseline toll-like receptor expression than the left ventricle—where sustained exposure promotes tricuspid valve endothelial activation and extracellular matrix degradation, thereby mechanistically linking hepatic inflammation to TR progression. In our analysis of AF patients with DBIL ≥3.5 µmol/L, those receiving antibiotic therapy demonstrated significantly reduced DBIL levels (Δ–1.8 μmol/L vs. controls, *p* = 0.007) and 32% lower risk of moderate-to-severe TR development (HR 0.68, 95% CI 0.51–0.89). This suggests that antibiotics disrupt the gut–liver–right heart axis by resolving intestinal inflammation, consequently attenuating both DBIL elevations and TR risk—directly explaining the observed correlation between elevated DBIL and TR severity in AF populations.

Serum DBIL functions as a biomarker of enterohepatic inflammation, where gut microbiota dysbiosis—particularly Streptococcus-driven endotoxin release—promotes hepatic Kupffer cell activation and systemic endotoxemia. Antibiotics attenuate this cascade by suppressing pathogenic bacterial translocation, thereby reducing inflammatory mediators that concurrently elevate DBIL and provoke tricuspid valve endothelial damage through right-heart cytokine exposure (13, 31, 32). Our observations in AF patients with elevated DBIL align with this mechanism: antibiotic users exhibited significantly lower progression to moderate-to-severe TR, most notably in cases involving Streptococcus-associated valvular pathology.

The observed TR risk reduction was restricted to the DBIL-high subgroup and reflects indirect associations derived from retrospective data. Unmeasured confounders such as variations in heart failure management or surveillance intensity may have contributed to this signal. Prospective validation will require intervention trials randomizing antibiotics vs. placebo in AF patients with confirmed TR and elevated DBIL, paired with microbial phenotyping of valvular vegetations and quantification of tissue-level inflammatory markers before and after therapy to establish causality (29, 30).

This study has a number of shortcomings. First, because this is a retrospective research, biases in information and selection may exist. Second, the limited sample size, particularly the imbalance between the large non-TR group (*n* = 253) and the smaller TR group (*n* = 92), reduces the statistical power of the analyses and limits the generalizability of our findings. Furthermore, because of this limited sample size, meaningful subgroup analyses (e.g., by specific antibiotic class or type of atrial fibrillation) could not be performed; investigating these relationships should be a focus of future research. Third, there are challenges arising from the fact that moderate-to-severe TR is multifactorial. Unmeasured variables have the potential to generate bias even when confounding factors have been thoroughly taken into account. Furthermore, a detailed prognostic analysis of serum DBIL in AF patients with moderate-to-severe TR was impeded by inadequate equipment availability, which hampered the collection of cardiac structural and hemodynamic data.

## 5 Conclusions

Elevated serum DBIL levels demonstrate significant diagnostic value for moderate-to-severe TR in patients with AF, with an optimal cutoff value of 3.5 μmol/L providing high sensitivity (91.2%) and specificity (68.8%). This correlation is further supported by the observed protective association of antibiotic therapy, potentially linked to anti-inflammatory mechanisms. Based on these findings, we propose that DBIL measurement should be integrated as an auxiliary screening tool into clinical practice, particularly for high-risk AF patients undergoing initial cardiovascular assessment or those with suspected right heart dysfunction. When DBIL levels exceed the 3.5 μmol/L threshold, this should prompt comprehensive echocardiographic evaluation to confirm TR severity, thereby enabling earlier intervention and optimized management strategies.

Future studies should focus on several key directions to validate and extend our findings. First, large-scale prospective multicenter trials are needed to confirm the diagnostic accuracy of DBIL (≥3.5 μmol/L) for moderate-to-severe TR in diverse AF populations and establish standardized clinical implementation protocols. Second, mechanistic investigations should explore the underlying pathways linking bilirubin metabolism to TR progression, including the role of gut–heart axis interactions, systemic inflammation, and endothelial dysfunction. Third, interventional studies examining how targeted antibiotic therapy or other anti-inflammatory approaches might modulate DBIL levels and TR outcomes would provide crucial insights into causal relationships. Finally, research should address whether DBIL monitoring following TR treatment (e.g., postsurgical repair) could serve as a dynamic biomarker for disease recurrence or therapeutic response, ultimately contributing to personalized medicine in AF-related valvular heart disease.

## Data Availability

The data that support the findings of this study are available on request from the corresponding author, Y-sW, upon reasonable request.

## References

[1] Rodrigues TS Quarto LJG Nogueira SC Koshy AN Mahajan R Sanders P Incidence and progression of atrial fibrillation in patients with and without heart failure using mineralocorticoid receptor antagonists: a meta-analysis. Clin Res Cardiol. (2024) 113(6):884–97. 10.1007/s00392-023-02349-3 38170251

[2] Li H Wang Y Yu C Wang C He Y Yang X Global, regional, and national burden of disease study of atrial fibrillation/flutter, 1990–2019. BMC Public Health. (2022) 22:2015. 10.1186/s12889-022-14403-2 36329400 PMC9632152

[3] Anagnostopoulos I Kousta M Kossyvakis C Paraskevaidis NT Schizas N Vrachatis D Atrial strain and occult atrial fibrillation in cryptogenic stroke patients: a systematic review and meta-analysis. Clin Res Cardiol. (2023) 112(11):1600–9. 10.1007/s00392-023-02218-z 37154833

[4] Sagris M Vardas EP Theofilis P Antonopoulos AS Oikonomou E Tousoulis D. Atrial fibrillation: pathogenesis, predisposing factors, and genetics. Int J Mol Sci. (2021) 23(1):6. 10.3390/ijms23010006 35008432 PMC8744894

[5] Hahn RT Zamorano JL Foster E Shah AN Asch FM. Tricuspid regurgitation: recent advances in understanding pathophysiology, severity grading and outcome. Eur Heart J Cardiovasc Imaging. (2022) 23(7):913–29. 10.1093/ehjci/jeac009 35157070

[6] Nguyen TKH Rudski LG. Optimal echocardiographic approach to the evaluation of tricuspid regurgitation. Curr Cardiol Rep. (2020) 22(9):108. 10.1007/s11886-020-01367-1 32770434

[7] Cho MS Park SJ Park JJ Shin J Lee JS Kim ES. Incidence and predictors of severe tricuspid regurgitation in atrial fibrillation patients without structural heart disease. Am J Cardiol. (2023) 203:288–94. 10.1016/j.amjcard.2023.07.005 37517122

[8] Patlolla SH Schaff HV Cavalcante JL Nishimura RA Stulak JM Chamberlain AM Incidence and burden of tricuspid regurgitation in patients with atrial fibrillation. J Am Coll Cardiol. (2022) 80(24):2289–98. 10.1016/j.jacc.2022.09.045 36480971

[9] Shevchenko OS Duzhiy GD Gutor SS. Functional state of the liver in pulmonary tuberculosis. Wiad Lek. (2023) 76(2):352–9. 10.36740/WLek202302116 37010173

[10] Ambrosy AP Vaduganathan M Huffman MD Khan S Kwasny MJ Fought AJ Clinical course and predictive value of liver function tests in patients hospitalized for worsening heart failure. Eur J Heart Fail. (2012) 14(3):302–11. 10.1093/eurjhf/hfs007 22357577

[11] Henein MY Vancheri S Longo G. The role of inflammation in cardiovascular disease. Int J Mol Sci. (2022) 23(23):12906. 10.3390/ijms232112906 36361701 PMC9658900

[12] Wang B Tontonoz P. Phospholipid remodeling in physiology and disease. Annu Rev Physiol. (2019) 81:165–88. 10.1146/annurev-physiol-020518-114444 30379616 PMC7008953

[13] Zhang Z Lv T Wang X Wu M Zhang R Yang X Role of the microbiota-gut-heart axis between bile acids and cardiovascular disease. Biomed Pharmacother. (2024) 174:116567. 10.1016/j.biopha.2024.116567 38583340

[14] Chen W Zhang S Wu J Ye T Wang S Wang P Butyrate-producing bacteria and the gut-heart axis in atherosclerosis. Clin Chim Acta. (2020) 507:236–41. 10.1016/j.cca.2020.04.037 32376324

[15] Ogawa M Nagata M Taniguchi Y Hasegawa H Yagi N Shiota T. Effect of right ventricular free wall longitudinal strain on all-cause death in patients with isolated severe tricuspid regurgitation and atrial fibrillation. Front Cardiovasc Med. (2023) 10:1188005. 10.3389/fcvm.2023.1188005 37808882 PMC10551442

[16] Maeder MT Holst DP Kaye DM. Tricuspid regurgitation contributes to renal dysfunction in patients with heart failure. J Card Fail. (2008) 14(10):824–30. 10.1016/j.cardfail.2008.07.236 19041045

[17] Aziz TM Burgess MI El-Gamel A Keevil B Rahman AN Campbell CS Clinical significance of tricuspid valve dysfunction after orthotopic heart transplantation. J Heart Lung Transplant. (2002) 21(10):1101–8. 10.1016/S1053-2498(02)00433-3 12398875

[18] Huang MJ Chen PL Huang CS. Bilirubin metabolism and UDP-glucuronosyltransferase 1A1 variants in Asians. Kaohsiung J Med Sci. (2022) 38(8):729–38. 10.1002/kjm2.12579 35942604 PMC11896460

[19] Petrtýl J Hůlek P Liberda M Hartmannová H Stanická S Piťha J Association of serum bilirubin and functional variants of heme oxygenase 1 and bilirubin UDP-glucuronosyl transferase genes. Antioxidants. (2021) 10(12):1964. 10.3390/antiox10122000 34943103 PMC8698489

[20] Yang X Liu L Zhang Y Hu H Gao B Liu T Inhibition of human UDP-glucuronosyltransferases 1A1-mediated bilirubin glucuronidation. Drug Metab Dispos. (2022) 50(5):552–65. 10.1124/dmd.121.000714 35241486

[21] Li Y Jia Y Wang Y Lin J Wang M Zhu J. Bilirubin stabilizes the mitochondrial membranes during NLRP3 inflammasome activation. Biochem Pharmacol. (2022) 203:115204. 10.1016/j.bcp.2022.115204 35944727

[22] Hariharan R Odjidja EN Scott D Shivappa N Hébert JR Hodge A The dietary inflammatory index, obesity, type 2 diabetes, and cardiovascular risk factors. Obes Rev. (2022) 23(3):e13349. 10.1111/obr.13349 34708499

[23] Toldo S Mauro AG Cutter Z Abbate A. Targeting the NLRP3 inflammasome in cardiovascular diseases. Pharmacol Ther. (2022) 236:108053. 10.1016/j.pharmthera.2021.108053 34906598 PMC9187780

[24] Yu Y Yan Y Niu F Wang Y Chen X Su G Ferroptosis: a cell death connecting oxidative stress, inflammation and cardiovascular diseases. Cell Death Discov. (2021) 7:193. 10.1038/s41420-021-00579-w 34312370 PMC8313570

[25] Stark K Massberg S. Interplay between inflammation and thrombosis in cardiovascular pathology. Nat Rev Cardiol. (2021) 18(10):666–82. 10.1038/s41569-021-00552-1 33958774 PMC8100938

[26] Karam BS Chavez-Moreno A Koh W Akar JG Akar FG. Oxidative stress and inflammation as central mediators of atrial fibrillation in obesity and diabetes. Cardiovasc Diabetol. (2017) 16:120. 10.1186/s12933-017-0604-9 28962617 PMC5622555

[27] Lau GT Tan HC Kritharides L. Type of liver dysfunction in heart failure and its relation to the severity of tricuspid regurgitation. Am J Cardiol. (2002) 90(12):1405–9. 10.1016/S0002-9149(02)02886-2 12480058

[28] Philip J Satyapal R Moodley J Thomas KE. Severe direct hyperbilirubinemia as a consequence of right heart failure in congenital heart disease. World J Pediatr Congenit Heart Surg. (2018) 9(4):470–4. 10.1177/2150135116640786 27154793

[29] Greabu M Logofatu C Tanase AM Papagheorghe LMI Radu C Giurcaneanu C. The levels of bilirubin may be related to an inflammatory condition in patients with coronary heart disease. Acta Pol Pharm. (2001) 58(3):225–31. 11712741

[30] Sarabi MM Abdollahi M Soukhtanloo M. Bilirubin and epigenetic modifications in metabolic and immunometabolic disorders. Endocr Metab Immune Disord Drug Targets. (2022) 22(11):1178–90. 10.2174/1871530321666211125102924 34823463

[31] Zoratti C Bottino R D’Errico S Cortinovis M Mariani F Brunetta E. Antibiotics and liver cirrhosis: what the physicians need to know. Antibiotics. (2021) 10(2):133. 10.3390/antibiotics11010031 35052907 PMC8772826

[32] Dong YH Chang CH Lin JW Wu LC Dormuth CR Lai MS. Association between use of fluoroquinolones and risk of mitral or aortic valve regurgitation. Clin Pharmacol Ther. (2024) 115(1):147–57. 10.1002/cpt.3084 37926942

